# Modulation by decitabine of gene expression and growth of osteosarcoma U2OS cells *in vitro *and in xenografts: Identification of apoptotic genes as targets for demethylation

**DOI:** 10.1186/1475-2867-7-14

**Published:** 2007-09-10

**Authors:** Khaldoun Al-Romaih, Gino R Somers, Jane Bayani, Simon Hughes, Mona Prasad, Jean-Claude Cutz, Hui Xue, Maria Zielenska, Yuzhuo Wang, Jeremy A Squire

**Affiliations:** 1 Department of Laboratory Medicine and Pathobiology, University of Toronto, Toronto, Canada. M5G 1L5; 2 The Ontario Cancer Institute, Princess Margaret Hospital, Toronto, Canada. M5G 2M9; 3 Department of Pediatric Laboratory Medicine, Hospital for Sick Children, Toronto, Canada. M5G 1X8; 4 Department of Cancer Endocrinology, British Columbia Cancer Agency, Vancouver, Canada. V5Z 1L3; 5 Departments of Pathology & Molecular Medicine, and Laboratory Medicine, McMaster University, St. Joseph's Healthcare – Hamilton Regional Laboratory Medicine Program, Hamilton, Canada L8N 4A6; 6 Division of Tumor Biology, Institute of Cancer and Cancer Research, UK Clinical Centre, Barts and the London School of Medicine and Dentistry, John Vane Science Centre, Charterhouse Square, London, United Kingdom, EC1M 6BQ; 7 The Prostate Centre, Vancouver General Hospital, Vancouver, Canada, V6H 3Z6

## Abstract

**Background:**

Methylation-mediated silencing of genes is one epigenetic mechanism implicated in cancer. Studies regarding the role of modulation of gene expression utilizing inhibitors of DNA methylation, such as decitabine, in osteosarcoma (OS) have been limited. A biological understanding of the overall effects of decitabine in OS is important because this particular agent is currently undergoing clinical trials. The objective of this study was to measure the response of the OS cell line, U2OS, to decitabine treatment both *in vitro *and *in vivo*.

**Results:**

Microarray expression profiling was used to distinguish decitabine-dependent changes in gene expression in U2OS cells, and to identify responsive loci with demethylated CpG promoter regions. U2OS xenografts were established under the sub-renal capsule of immune-deficient mice to study the effect of decitabine *in vivo *on tumor growth and differentiation. Reduced nuclear methylation levels could be detected in xenografts derived from treated mice by immunohistochemistry utilizing a 5-methylcytidine antibody. Decitabine treatment reduced tumor xenograft size significantly (p < 0.05). Histological analysis of treated U2OS xenograft sections revealed a lower mitotic activity (p < 0.0001), increased bone matrix production (p < 0.0001), and a higher number of apoptotic cells (p = 0.0329). Microarray expression profiling of U2OS cultured cells showed that decitabine treatment caused a significant induction (p < 0.0025) in the expression of 88 genes. Thirteen had a ≥2-fold change, 11 of which had CpG-island-associated promoters. Interestingly, 6 of these 11 were pro-apoptotic genes and decitabine resulted in a significant induction of cell death in U2OS cells *in vitro *(p < 0.05). The 6 pro-apoptotic genes (*GADD45A*, *HSPA9B*, *PAWR*, *PDCD5*, *NFKBIA*, and *TNFAIP3*) were also induced to ≥2-fold *in vivo*. Quantitative methylation pyrosequencing confirmed that the tested pro-apoptotic genes had CpG-island DNA demethylationas a result of U2OS decitabine treatment both *in vitro *and in xenografts

**Conclusion:**

These data provide new insights regarding the use of epigenetic modifiers in OS, and have important implications for therapeutic trials involving demethylation drugs. Collectively, these data have provided biological evidence that one mode of action of decitabine may be the induction of apoptosis utilizing promoter-CpG demethylation of specific effectors in cell death pathways in OS.

## Background

Epigenetic modifications involve genomic methylation changes and the alteration of chromatin-associated proteins such as linker histones, polycomb groups, nuclear scaffold proteins and transcription factors, (reviewed in [1]). Epigenetic abnormalities contribute in several ways to oncogenesis and may activate oncogenes or silence tumor suppressor genes. In addition, epigenetic processes can enhance chromosomal instability [2], (reviewed in [3]), and have recently been shown to be involved in the regulation of the DNA double-strand break and repair process [4], (reviewed in [5]). Typically, a general pattern of demethylation of the genome is observed in tumor DNA, while increased methylation of a subset of promoter-associated CpG islands associated with the transcriptional start sites (TSS) of genes may also be observed. Abnormal methylation of genes is far more frequent than classical genetic mutation.

There is increasing interest in the therapeutic modulation of such processes, since epigenetic alterations are amenable to physiological alteration by drugs that change patterns of DNA methylation or histone acetylation, (reviewed in [6]). The most powerful DNA methyltransferase inhibitor in clinical use is 5-aza-2'-deoxycytidine (decitabine). Decitabine is a cytosine analog that inhibits DNA methylation and reactivates silenced genes. Decitabine has shown promising clinical efficacy in the treatment of myelodysplastic syndromes, with evidence of gene target expression modulation by demethylation with less toxicity than conventional cancer chemotherapies [7,8]. Studies regarding the implications of epigenetic modification in osteosarcoma (OS) have been limited, but have suggested a role in bone differentiation [9-11], transcription factor expression, and histone modifications [12,13]. No study to date has utilized decitabine to modify gene expression in an OS-derived cell to identify gene-specific targets for demethylation that may have therapeutic importance.

A full assessment of tumor cell response to treatment requires integrating experimental data from both *in vitro *and *in vivo *observations. One major advantage of preclinical animal models of xenografts of human tumor cell lines is that they provide both tissue vascularization and a tumor microenvironment that is closer to human tumors so that an evaluation of the therapeutic impact on tissue differentiation, cell growth and proliferation levels is possible [14], (reviewed in [15]). Such analyses are providing opportunities for a detailed assessment of new classes of anti-neoplastic drugs that target the epigenome, such as decitabine.

## Results

### Effects of decitabine on U2OS cells in vitro

**Figure 1:**
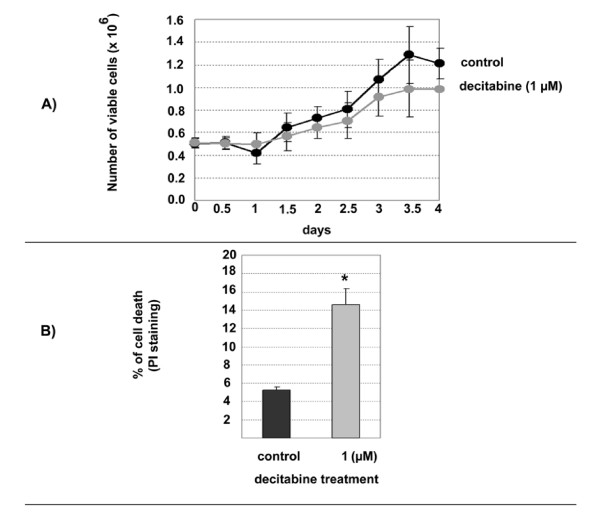
**Viability and growth kinetics of U2OS *in vitro***. **A)**, Growth curves of U2OS cells with growth medium alone (dark line), or growth medium with one dose of decitabine at a final concentration of 1 μM (light graph). The y-axis indicates cell number in millions and the x-axis indicates time points in days. The results are expressed as cell counts at each corresponding time point. Each data point is the Mean of cell counts from 2 experiments (5 passages apart) each consist of 2 independent cultures and the error bars indicate the standard deviation. The findings indicate a slight increase of U2OS cells' doubling time and a decrease of 18% (p = 0.045) in the viability of treated cells compared to untreated control **B)**, Cell death in U2OS cells caused by decitabine treatment at 1 μM concentration (light column) compared to no-treatment (dark column). The results are expressed as percentage of cell death (fraction of cells with positive PI stain). The y-axis indicates the percentage of cells with PI staining (dead cells). Each column is the Mean of 3 experiments with error bars indicating standard deviation. The asterisk indicates significant increase in cell death (p < 0.05) as a result of decitabine treatment.

Decitabine is a cytotoxic agent in addition to being a demethylation agent. U2OS growth rate was measured in the absence, or presence of 1 μM decitabine. This relatively low-dose was chosen to minimize cytotoxicity. The doubling time for U2OS in the absence of 1 μM decitabine was close to 2.75 days (66 hours) while the decitabine treated U2OS cells doubled at 3.5 days (84 hours). This was accompanied by a minor reduction in viable cell numbers as a result of decitabine treatment (Figure 1A). To confirm that decitabine was also causing DNA demethylation of specific loci we examined the extent of loss of the methylation known to characterize the small nuclear ribonucleoprotein polypeptide N (*SNRPN*) gene. This heavily methylated gene has previously been shown to undergo reduction in methylation following treatment with decitabine [16]. After 72 hours treatment of U2OS with 1 μM decitabine, ~60 % more DNA could be digested with a methylation-sensitive restriction endonuclease [see Additional file 1]. To determine whether a slower growth rate was accompanied by higher level of cell death following decitabine treatment, death assessment was studied by flow cytometry after PI staining. The control (no-treatment) cells showed a 5% death rate whereas the decitabine treatment significantly increased the death rate of 10% more than the control at day 3 (p < 0.05) (Figure 1B).

### Effects of decitabine on U2OS in vivo

**Figure 2:**
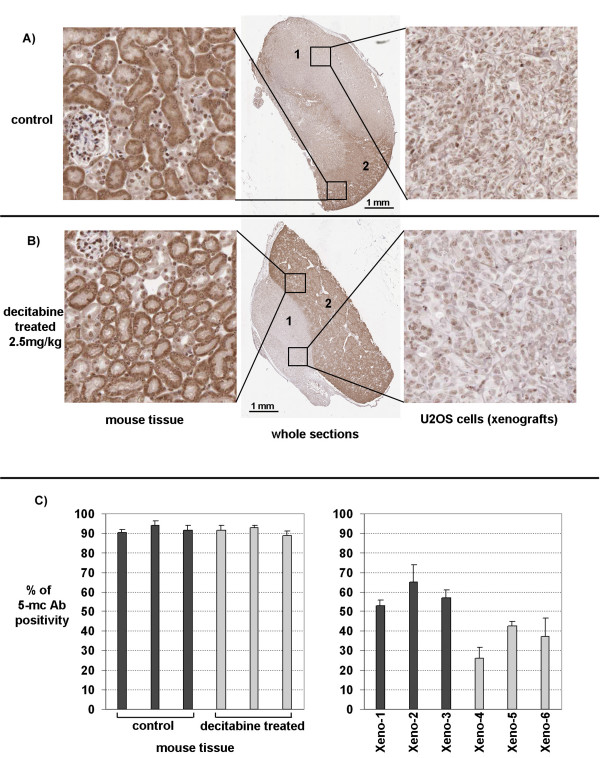
**Analysis of differential 5-methylcytidine content of U2OS xenografts using histological sections**. Analysis of the relative levels of cellular 5-methylcytidine within xenografts derived from representative control untreated mice (panel A); or decitabine treated mice (panel B) using immunohistochemical staining with 5-mc-Ab. To the right a 20X enlargement of representative U2OS histology is shown. More 5-mc-Ab staining is evident in the nuclei from control sections (panel A-right side) in comparison to nuclear staining in the sections derived from decitabine treated mice (panel B right side). These data are consistent with a reduction in 5-methylcytidine nuclear content in U2OS xenografts derived from mice treated with decitabine. Enlargements of darkly stained host murine kidney cells correspond to heavily methylated differentiated renal tissue (denoted 2 in panels A and B). In panel C the staining intensity from control (dark columns) and decitabine treated (light columns) is quantitated using Aperio scanning image analysis of sections. The graph to the right of panel C confirms that the 5-mc-Ab staining in control untreated (dark columns) U2OS xenograft sections is more intense than the decitabine treated (light columns) U2OS xenografts. This decrease in staining intensity was significant (p < 0.05). In contrast host kidney cells from both control and treated mice do not exhibit any significant difference in staining intensities.

U2OS has been shown to be generally non-tumorigenic using heterotopic grafts under the mouse skin [17]. In contrast the sub-renal capsule site has proved to be an excellent grafting site for xenografts in general [18-20]. Therefore U2OS xenografts were obtained from mice and cut into pieces of ~4 mm^3 ^and 24 tumor tissue pieces were regrafted under the renal capsules of 6 SCID mice (4 grafts per mouse). Four weeks after grafting the host mice were divided into two groups. One group of three mice was treated with Decitabine (2.5 mg/kg) intraperitoneally on Days 29, 31 and 33. The other group of three mice was given saline as control at the same schedule. On Day 37, all 6 mice were sacrificed and xenografts were dissected from the mice, and the tumor volumes were measured and compared in both treatment arms. The effectiveness of demethylation *in vivo *was determined by analyzing the relative cellular distribution of 5-methylcytidine levels using a specific 5-methylcytidine antibody and 6 tissue sections from 6 xenografts (3 control and 3 decitabine treated). In these analyses, nuclei in host kidney stromal cells were used as internal positive controls (see dark staining nuclei Figure 2). Negative controls obtained by omitting the primary antibody were also performed. Nuclear xenograft tumor staining was recorded by a semiquantitative and visual grading, considering both the intensity of staining and the proportion of positive tumor nuclei in the selected sections. Xenograft derived from the untreated control mice exhibited stronger nuclear staining than the staining present in nuclei from xenografts in decitabine treated mice (p < 0.05). These results were consistent with a widespread loss of methylation in U2OS xenografts derived from the treatment group.

**Figure 3:**
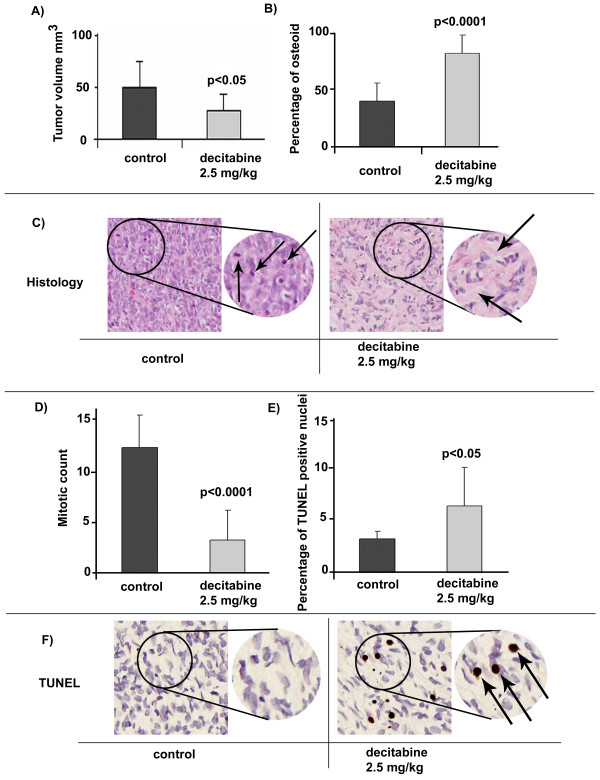
**Induction of differentiation and apoptosis by decitabine treatment of U2OS *in vivo***. **A)**, Decitabine effects on tumor volumes *in vivo*. The results compare tumor volumes in control xenografts to decitabine treated xenografts as described in methods. Each column is the Mean of tumor volumes measured in 12 xenograft tumors. There was a significant decrease (p < 0.05) in tumor volumes as a result of decitabine treatment. **B)**, Osteoid assessment *in vivo*. Each column is the Mean of osteoid evaluation of 9 sections. There was significant increase (p < 0.0001) in osteoid formation as a result of decitabine treatment. **C)**, Representation images of H&E sections. Control xenograft tumors (left) show solid sheets of poorly differentiated cells with minimal osteoid (image magnification × 100). Decitabine treated tumors  shows less dense cell population and increased areas of osteoid seen as light pink and lacy matrix with the nuclei of osteoblasts sitting closer to the produced matrix (image magnification × 100). The arrows in the enlargement  show the osteoid matrix surrounding osteoblasts (defined as eosinophilic osteoid-like material). **C), **And **D)**, Mitotic count as identified in the same sections used to asses for osteoid evaluation. Each column is the Mean mitotic count in 9 sections (a minimum of 1100 nuclei were scanned per section). Decitabine treatment resulted in a significantly lower mitotic count (p < 0.0001). The arrows in the enlargement image in **C **(left) indicate mitotic nuclei. **E)**, Results for apoptosis analysis by TUNEL assay. Each column is the Mean count of TUNEL positive nuclei seen in 9 images representing 9 sections (≥1000 nuclei were scanned per section). There was a significant increase (p < 0.05) of apoptotic cells as a result of decitabine treatment. **F)**, Representation images from TUNEL assay of control tumors (left) and decitabine treated tumors [50] (image magnification × 200). The arrows in the enlargement image show the TUNEL-positive nuclei (apoptotic nuclei). Error bars indicate standard deviation from the Mean values and p-values are based on comparison between control and decitabine treated tumors using student t-test.

Tumor volumes were determined for all 24 xenografts [see Additional file 2]. The average volume size for the xenografted tumors in the control group was 49 mm^3 ^(± 25SD). The xenograft tumors from the decitabine treated group had an average volume size of 27 mm^3 ^(± 15.9SD) indicating a significant decrease (p = 0.0096) of tumor volume as a result of the decitabine treatment (Figure 3A). All U2OS xenografts were whitish in color and the texture of tissues from both treatment groups had a moderate-hard consistency, with no apparent kidney tissue invasion.

Comparison between the U2OS xenograft histology from the decitabine-treated mice and the control mice identified differences in bone matrix (osteoid) content. In these studies relative levels of osteoid were determined in 9 sections each from the control and decitabine treatment groups. As shown in Figure 3B, histological analysis of the stained xenograft sections identified an overall average osteoid area of 42% in the control group. The decitabine treated group, however, had a significant increase (p < 0.0001) in osteoid formation reaching up to an average of 86%. With respect to tissue morphology, the tumors from the control mice showed solid sheets of poorly differentiated cells associated with a small amount of osteoid (Figure 3C left panel). In contrast, the xenograft tumors from the decitabine-treated mice showed a less dense cell population with increased areas of osteoid, seen as light pink and lacy matrix with the nuclei of osteoblasts sitting close to the matrix, a feature of differentiated osteoblasts in normal bone (Figure 3C right panel). The mitotic count was assessed in the same sections (Figure 3D). The mitotic index showed an average of 11.9 mitotic figures in the control group and an average of 3.1 mitotic figures in the decitabine treated group (p < 0.0001).

Since decitabine induced a higher level of cell death *in vitro *and there was a marked decrease in the size of U2OS derived tumors *in vivo*, the level of apoptotic cells in the xenograft tumors was analyzed using the TUNEL assay. Scoring was performed on digital images from 9 control sections and 9 decitabine treatment sections. The average apoptotic indices were 3.2% in the control group and 6.2% in the decitabine treatment group (p = 0.0329) (Figure 3E and 3F).

### Expression profiling of decitabine-induced changes in gene expression

Expression profiling of 14,500 transcripts with known gene function was performed using the Affymetrix U133A microarrays following treatment of U2OS cells with 1 μM decitabine. Eighty-eight (88) genes [see Additional file 3] exhibited significant (p < 0.0025) up-regulation after two independent treatments of U2OS with 1 μM decitabine. Within this large group of 88 genes there were 13 with a ≥2-fold change in both experiments (Table 1). The expression of subset of 7 of these robustly reactivated genes was examined by real-time PCR and increased gene expression was consistent with the levels detected by microarray analysis (Table 1).

**Table 1:** Genes up-regulated (p < 0.0025 and ≥2 fold-change) after decitabine treatment identified using AffyChip.

**Gene Symbol**	**Fold Change (Affy)**	**Fold Change (Real-Time)**	**CpG**	**known or proposed function**
FAM65A	2.76	nd	yes	hypothetical protein LOC79567
**GADD45A**	**2.58**	**6**	**yes**	**Regulation of cyclin dependent protein kinase activity and DNA repair**
GAGE4	3.51	nd	yes	Unknown
**HSPA9B**	**2.32**	**5.3**	**yes**	**Implicated in the control of cell proliferation and cellular aging**
IGFBP6	2.22	3	yes	Insulin-like growth factor binding protein involved in negative regulation of cell proliferation
IMP-3	8.36	nd	yes	Binds to insulin-like growth factor II leader 3 mRNA and may repress its translation during late development
**NFKBIA**	**4.01**	**4.2**	**yes**	**Inhibits NF-k-B and involved in cell adhesion, apoptosis, differentiation and growth**
**PAWR**	**3.30**	**10**	**yes**	**Pro-apoptotic protein acts by down-regulating the anti-apoptotic protein BCL2 via its interaction with WT1**
**PDCD5**	**4.15**	**8**	**yes**	**Induction of programmed cell death**
PSG5	2.32	nd	no	Synthesized by syncitiotrophoblast of the placenta and has a role in maintenance of gestation
RAC2	5.09	nd	no	Involved in the regulation of the NADPH oxidase
TGM2	2.01	nd	yes	A peptide cross-linking factor
**TNFAIP3**	**3.44**	**4.5**	**yes**	**Negative regulation of I-k-B kinase/NF-k-B cascade**

Fold change (Affy) is the Mean from a duplicate AffyChip experiments. The fold change using real-time PCR (TaqMan method) is the Mean values from the same RNA from the same yields that were used for the AffyChip experiments and a third replica. The direction of expression was consistent in the Affy experiment and the real-time experiment for the tested genes. 11 out of 13 genes have CpG-island in there 5' region. Genes in bold characters are those identified in the apoptotic pathway as explained in "Methods" and "Results". The genes are listed in alphabetical order.

An *in silico *analysis of CpG islands associated with the 88 up-regulated genes was then performed. 63 genes (71%) were found to contain CpG-islands within their 5'-regulatory region. Of the 13 genes with a ≥2fold-change, 11 genes (84%) had a CpG-island within their 5' region (Table 1). Pathway enrichment analysis was performed on the 88 up-regulated genes, and the three top enriched pathways included: "negative regulation of cellular processes" (p = 0.007), "positive regulation of programmed cell death" (p = 0.01) and, "organelle organization and biogenesis" (p = 0.03). Interestingly, when the 13 genes with a ≥2-fold change were analyzed in the same manner, six genes were assigned to the "apoptosis pathway" with high significance (p < 0.0001). These genes include *GADD45A*, *HSPA9B*, *PAWR*, *PDCD5*, *NFKBIA*, and *TNFAIP3*, which were selected for expression validation in the xenografts and in normal human osteoblasts (NHOst).

### Real-Time expression analysis for apoptotic genes in U2OS xenografts and normal osteoblasts

**Figure 4:**
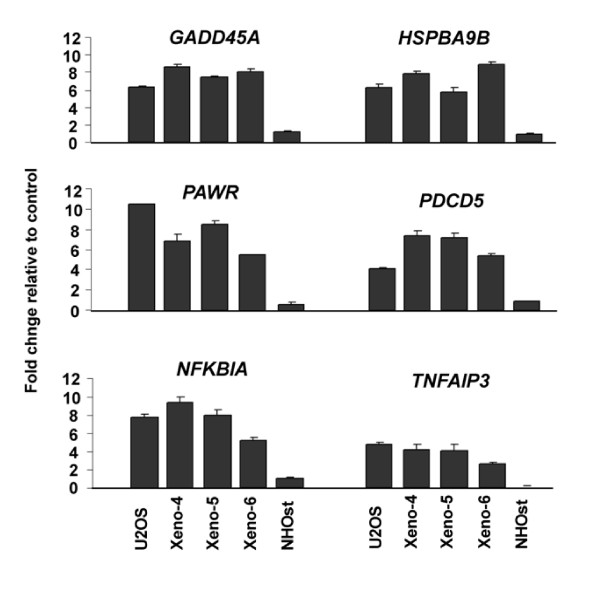
**Real-time gene expression of 6 pro-apoptotic genes U2OS *in vitro *&*in vivo *and normal osteoblasts**. Total RNA was extracted from U2OS cells *in vitro *and *in vivo *and reverse-transcribed as detailed in methods. TaqMan assays were used to determine relative expression using the cDNA from control (no treatment) as base lines and *ACTB *for a reference gene by applying the ΔΔCt method. Each column is the Mean of three replicas and error bars indicate standard deviation from the Mean. The data is expressed as fold change relative to control (no-treatment). Xeno-4, Xeno-5, and Xeno-6 = decitabine treated xenografts. NHOst = normal human osteoblasts.

Real-time PCR (TaqMan method) was used to validate expression of the apoptotic genes shown in Table 1 in the xenografts and NHOst. As illustrated in Figure 4, the change of expression was expressed as fold change relative to the control (no treatment), using beta-actin (*ACTB*) as a reference. Up-regulation, as a result of decitabine treatment was consistent in U2OS cells *in vitro *and *in vivo*. Gene expression of the apoptotic genes was up-regulated up to 8-fold for *GADD45A*, 8-fold for *HSPA9B*, 12-fold for *PAWR*, 8-fold for *PDCD5*, 9-fold for *NFKBIA*, and 5-fold for *TNFAIP3*. In addition, real-time expression for the six genes was examined in normal osteoblasts before and after treatment with decitabine. There was marginal increase of expression after treatment (<2-fold change) in normal osteoblast (Figure 4).

### DNA methylation analysis of decitabine responsive genes using Pyro Q-CpG

**Figure 5:**
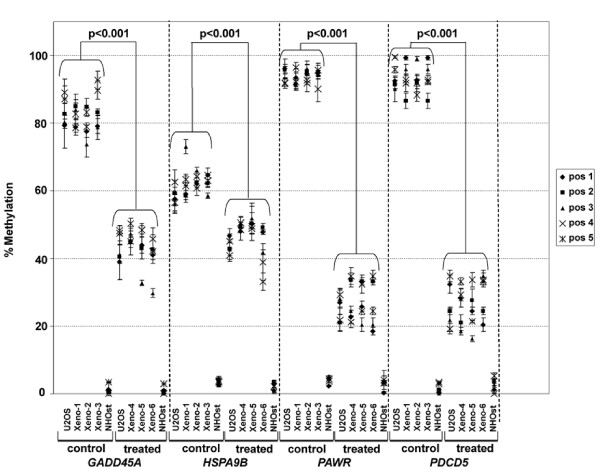
**Comparison of percentage of methylation across 5 of the tested CpG positions *in vitro *and *in vivo***. Each data point is the average of methylation percentage for each CpG position in three experiments. Y-axis indicates the percentage of methylation and the samples are indicated on the x-axis. The location of CpG positions relative to the gene start site and to each other is shown in Figure 5. The results of 5 CpG positions are shown to represent the methylation percentage in the four genes across the samples. There was a significant decrease (p < 0.001) in methylation quantity for each CpG position after decitabine treatment both *in vitro *and *in vivo *for the four genes in all sample but not in the NHOst (normal human osteoblasts). p-values were calculated by comparing the percentage of methylation for each individual CpG position in control cells with the same CpG position in the treated cells using student t-test and they all resulted in p < 0.001.

Methylation Pyro Q-CpG sequencing analysis was performed on six genes with expression up-regulation after decitabine treatment including *GADD45A*, *HSPA9B*, *PAWR*, *PDCD5*, *NFKBIA*, and *TNFAIP3*. *GADD45A*, *HSPA9B*, *PAWR*, and *PDCD5*, but not *NFKBIA*, and *TNFAIP3*, showed reliable and reproducible results for the tested amplicons. *GADD45A*, *PAWR*, and *PDCD5 *had a high methylation content (>70%) without decitabine treatment, while *HSPA9B *had an intermediate (50% – 70%) methylation content. Decitabine treatment lowered the methylation content in all CpG sites tested for the four genes *in vitro *and *in vivo *(Figure 5). Induction of demethylation was most marked for *PAWR*, and *PDCD5*. *GADD45A *had intermediate loss of methylation and *HSPA9B *had the least change but overall methylation loss remained significant. Analysis of relative change in methylation was also performed on normal osteoblasts, which had an initial low methylation content comparable to the negative control (DNA from early embryos), and no change in this basal content was apparent following decitabine treatment.

**Figure 6:**
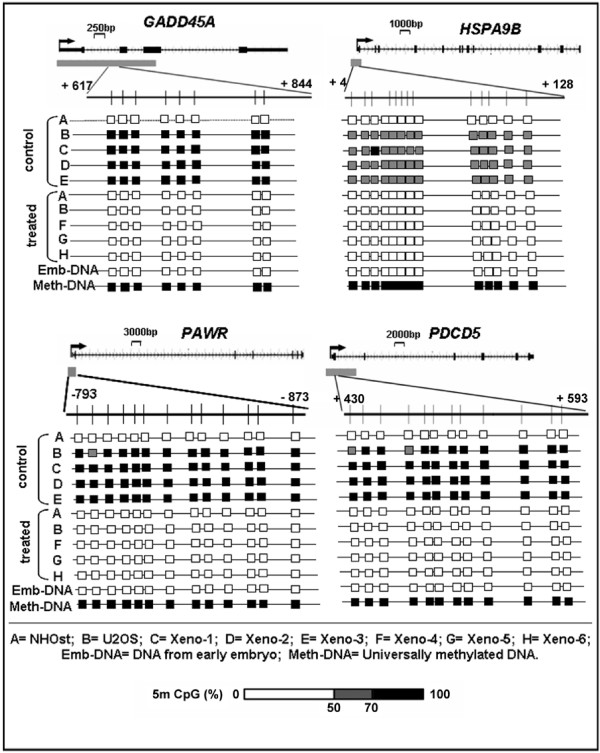
**Summary of Pyro-Q-CpG findings**. Illustrated are the summary findings by Pyro-Q-CpG analysis of U2OS cells *in vitro *without treatment (control) and with 1 μM decitabine treatment (treated). The illustration also summarizes Pyro-Q-CpG findings in control U2OS xenograft tumors (Xeno-1, Xeno-2, and Xeno-3), and 2.5 mg/kg decitabine treated U2OS xenograft tumors (Xeno-4, Xeno-5, and Xeno-6). DNA from NHOst (normal low-passage human osteoblasts) was also analyzed for experiment control. DNAs from early embryonic DNA (Emb-DNA) and universally methylated DNA (Met-DNA) were used for negative and positive control respectively. CpG-islands are denoted by grey rectangles relative to the gene start site. The region further enlarged below corresponds to each tested CpG sequence. The tick marks denotes the individual CpG dinucleotides. The transcription start site is indicated by a directional arrow with the base pair numbers annotated for each tested sequence. The extent of methylation is represented by the scale bar (bottom right). *GADD45A*, *PAWR*, and *PDCD5*, had a high level of methylation before decitabine treatment while *HSPA9B *had an intermediate level of methylation before treatment. In all cases the methylation was decreased significantly (p < 0.001) as a result of decitabine treatment. The detailed results for all genes are shown in [Additional file 4].

More detailed results on the methylation status for the same four genes for all tested CpG positions is provided in Figure 6 [also see Additional file 4]. For *GADD45A*, a CpG rich sequence at the promoter region containing a cluster of eight CpG positions was tested by Pyro Q-CpG. In U2OS cells, the methylation percentage on the eight CpG positions had an average of 83% in the no-treatment (control) cells. This was reduced to an average of 44% after treatment with 1 μM decitabine (p < 0.001). Similarly, the no-treatment xenograft tumors had averages of 84% in Xeno-1, 81% in Xeno-2, and 85% in Xeno-3, which was reduced after three doses of 2.5 mg/kg decitabine to 46%, 43% and 37% in Xeno-4, Xeno-5 and Xeno-6 respectively (p < 0.001).

A CpG rich sequence at the promoter region of *HSPA9B *had a cluster of 13 CpG positions which were tested by Pyro Q-CpG. The overall methylation quantity seen in this sequence was lower than what was observed in *GADD45A*, none-the-less the difference between the no-treatment (control) samples and decitabine-treated samples was significant (p < 0.001). U2OS cells (*in vitro*) had an average of 59% before treatment, and 42% after treatment (p < 0.001). Before treatment, the xenograft tumors had averages of 61% in Xeno-1, Xeno-2, and Xeno-3 which was reduced after treatment to an average of 49% in Xeno-4 and Xeno-5 and 43% in Xeno-6 (p < 0.001).

The analysis was done in the same manner for 13 and 12 CpG positions related to the CpG-island associated with *PAWR *and *PDCD5 *respectively. These two genes had very high methylation percentage before decitabine treatment in U2OS, Xeno-1, 2, and 3 for the tested CpG positions. In the case of the 13 CpG positions tested in *PAWR *the average of methylation percentage before decitabine treatment was 93% in U2OS cells, 93% in Xeno-1 and Xeno-2, and 94% in Xeno-3. After decitabine treatment, this was reduced significantly (p < 0.001) to an average of 26% in U2OS cells, 29% in Xeno-4 and 28% in Xeno-5 and 6. In the 12 CpG positions tested in *PDCD5*, U2OS cells had an average of 93%, Xeno-1 had an average of 94, Xeno-2 had an average of 93%, and Xeno-3 had an average of 92% before decitabine treatment. The methylation was reduced significantly after decitabine treatment (p < 0.001) to 26% in U2OS cells, Xeno-4, and Xeno-5, and 27% in Xeno-6.

The normal low-passage osteoblast had a very low methylation percentage in all 4 genes. The range of methylation percentage had averages from 2.1% to 3.6% before treatment, and 1.4% to 2.7% after treatment with 1 μM decitabine across all the tested CpG positions in the 4 genes (Figure 5, Figure 6 and [Additional file 4]). Importantly, the methylation status of the tested CpG sequences reflects the patterns of expression seen in all four genes after decitabine treatment in U2OS cells and xenografts. This is consistent with the possibility that decitabine treatment modulated the expression through reducing the amount of methylation on CpG-dinucleotides. To determine whether decitabine activated *GADD45A *methylation status and expression was also tested in two other OS cell lines, MG63 and HOS using identical decitabine treatment conditions. Similar to U2OS, induction of *GADD45A *gene expression was associated with significant loss of methylation and increased transcript expression in MG63 cells. However in HOS cells, treatment had no significant demethylation effect on the *GADD45A *promoter regions and also failed to activate expression of the gene (data not shown).

## Discussion

This study draws attention to the possibility that therapeutic levels of decitabine could orchestrate the interplay between DNA damage genes, induce growth arrest, apoptosis and potentially modulate genomic fidelity. At present, neoadjuvant and adjuvant chemotherapy is favored in the treatment of OS and the agents most commonly used include doxorubicin, high-dose methotrexate, cis-platinum and ifosfamide either alone or with etoposide. The use of these agents in OS treatment has been well established and yielded 5-year disease-free-survival and overall-survival of greater than 60%, (reviewed in [21]). However, the lack of a near-complete response to chemotherapy in a sub-group of patients reflects inherent biologic resistance to these agents, hence poorer prognosis [22], especially since attempts at changing chemotherapy regimens for poor responders have generally not improved outcome [23-25]. In recent years, another approach that has been used in other tumors is targeting the epigenome of tumor cells. The most promising of which is the re-activation of epigenetically silenced genes, using DNA methylation inhibitors or histone deacetylase inhibitors (HDAC) [26-28].

Previous studies have implicated a role of epigenetics in OS biology; Methylation of osteocalcin has been linked to bone differentiation [9-11], transcription factor expression, and histone modifications [12,13]. Abnormal promoter methylation of p16INK4a/p14ARF promoters was observed in OS-derived cell lines [29]. Aberrant methylation of specific genes was also correlated with poor survival in OS patients [30,31]. The number of genes found methylated in OS is increasing [32,33] which further supports the implication of DNA methylation in OS tumorigenesis.

Up-to-date studies regarding the epigenetics OS have either been based on a single gene or focused on a small number of genes, and limited with respect to elucidating the target pathways suitable for epigenetic therapeutics in OS. Our study is the first to use demethylation treatment to modify global gene expression in an OS cell line in order to identify pathway-specific methylation targets that may have therapeutic importance. Analysis of decitabine-induced cellular changes in U2OS xenografts suggested that apoptotic pathways may be the earliest pathways to be affected. The decitabine dose was based on previous studies [34-36] and has been shown to reduce the methylation of tumor suppressor genes and decrease tumor growth in xenografts. The xenografts in decitabine-treated mice decreased in volume size significantly (p < 0.05) when compared to the xenografts in untreated control mice. Recent reports showed similar effects of xenograft size and growth parameters from other tumor types grown in mice treated with decitabine [37,38].

The effect of decitabine on tumor cell mitotic index, apoptosis and extracellular matrix (osteoid) formation in OS has not been previously reported. In our series, decitabine treatment significantly decreased the number of mitoses and increased the number of apoptotic cells. In addition, treatment with decitabine significantly increased the amount of osteoid associated with the tumors. Such results suggest that decitabine treatment reduces the proliferative capacity of U2OS cells, whilst concurrently driving the cells toward terminal differentiation and apoptosis.

Gene expression profiling by microarray analysis showed that 88 genes showed increased expression *in vitro *after decitabine treatment, which represented 0.6% out of the 14500 genes on the array and this global modulation of genes is at a level comparable to other studies. The effect of decitabine on global gene expression has been previously reported in several studies [39-41]; comparable to our study, the number of up-regulated genes varied from 1.9% in ovarian cancer cells [40] to 1.1% in malignant glioma cells [41] and 0.6% in bladder cancer cells [39]. The differences in the number of decitabine up-regulated genes were possibly a result of cell line-to-cell line variations, microarray platforms, and experimental designs including differences in drug dosage and duration of treatment. Indeed, Karpf et al [42] screened the expression of approximately 38,000 human transcripts in several decitabine treated cell types and observed changes in genes expression for 0.2 to 1.4% of those transcripts, depending on the cell type treated [42] and (reviewed in, [43]). Further investigation of several OS cell lines will further assist to more accurately define the level of global change of expression induced by decitabine using the same experiment conditions and microarray platform.

The global expression profiling of U2OS represents the majority of cells that were viable at the time of harvest. These cells were undergoing fine molecular modulations of their epigenome eventually leading to changes in gene expression resulting in the observed cell death and apoptosis *in vitro *and *in vivo*. Decitabine treatment in U2OS induced a level of cell death that was comparable to observations in acute myeloid leukemia (AML) cells using similar treatment regimens *in vitro *[44]. In this study differentiation activation was considered to be an early effect of decitabine in AML. The genes identified in U2OS with a role in apoptosis were *GADD45A*, *HSPA9B*, *PAWR*, *PDCD5*, *NFKBIA*, and TNFAIP3. These proteins have potential roles in regulating a number of key apoptotic events including the p53 related apoptosis, bcl2 related apoptosis, and the nfkb related apoptosis [40-50]. While the exact function of *GADD45A *is not known, the protein, like p53, is considered to be involved in cell growth control, maintenance of genomic stability, DNA repair, cell cycle control, and apoptosis. Interestingly, Gadd45a has recently been shown to promote epigenetic gene activation by repair-mediated DNA demethylation [45].

The findings of this study suggest that the cellular effects detected are a result of up-regulation of apoptotic genes. These data are in general agreement with the increasing evidence that decitabine's antineoplastic effects may be through modulation of apoptotic pathways [46,47]. Our data also demonstrate that the re-activation of genes involves CpG-island demethylation. Dinucleotide clusters of CpGs in CG-rich regions of genomes or CpG-islands are present in the promoters and exonic regions of at least 40% of mammalian genes some reports, however, other reports indicate that up to 70% of mammalian genes have CpG islands in their promoter [48,49]. Methylation of promoter associated CpG islands in the genome of cancer cells has shown non-random and tumor-type-specific patterns [50]. While some tumors exhibit hypermethylation of low number of specific CpG islands, other tumors possess hypermethylation of a higher number CpG island associated promoters [50]. It is important, though, to realize that not all genes with methylated CpG islands are re-activated by decitabine treatment possibly because some methylated CpG islands may have other chromatin structural alterations that are dominate over DNA methylation for their silencing, (reviewed in [43]).

In our study, 63 (71%) of the 88 decitabine up-regulated genes possessed CpG-island at their 5' region, a proportion that is higher than that observed in other reports; including AML [44], and human glioma cell lines, where decitabine re-activated 50% and 40%, respectively, of genes with potential CpG islands [50]. This observation is particularly intriguing because it suggests that decitabine treatment of U2OS induced CpG-island associated genes more frequently than previously reported. Moreover, of the 13 U2OS genes with a ≥2-fold change, there were 11 genes (84%) with CpG-island in there promoter region. The further enrichment of the frequency of CpG-island-associated genes in the genes with a strong induction of expression (≥2-fold change) after decitabine treatment, further suggested that expression induction reflects either a more direct effect of decitabine through CpG-island demethylation, or indirect activation effects. In four of six apoptotic genes studied in detail, we showed a significant increase in expression following decitabine treatment was accompanied by a marked loss of promoter methylation, which points out to the potential direct effect of decitabine on methylated CpG sequences. Decitabine-induction of *GADD45A *expression was examined in two other osteosarcoma cell lines (MG63 and HOS), to determine the specificity of this effect. Whilst MG63 responded in a similar way to U2OS, HOS did not become demethylated in the promoter region of *GADD45A*, and expression was not activated by decitabine. These data are consistent with cell-type differences in response to this drug (Al-Romaih et al., in preparation), and the notion that mechanisms other than demethylation may effect a subset of genes in some cell types. Interestingly, out of 88 significantly induced genes there were 25 genes with no apparent CpG island at their promoter region. Similarly, two genes out of the 13 genes with ≥2-fold change have no potential CpG islands close to the TSS and promoter region. Methylation-independent induction of gene expression has also observed in other studies [39,51,44] indicating that genes without CpG islands may respond to this drug. Decitabine mechanism of action is not restricted to its demethylation capability and was reported to have effects on histone methylation and RB phosphorylation [52,53]. This also draws attention to the possibility that the other two apoptotic genes (*NFKBIA*, and *TNFAIP3*) were expressed by induction of methylation-independent mechanisms.

Decitabine treatment re-activated several apoptotic genes in U2OS cells that were identified in this work. Our microarray screen also identified other decitabine re-activated genes with potential roles in regulating proliferation and differentiation of mammalian cells. IGF-binding proteins IGFBP6 and IMP-3 (Table 1) have been shown to be potential regulators of IGFs with anti-growth properties [54-57]. Hypermethylation of these genes functions as a mechanism for increased proliferation capacity, reduction of apoptosis and loss of the differentiated phenotype in U2OS. Targeting hypermethylation in U2OS by decitabine indicated the potential power of this drug for OS treatment.

## Conclusion

Our data suggest that a number of pro-apoptotic genes were silenced by methylation in U2OS. Hypermethylation of *GAD45A*, *HSPA9B*, *PAWR*, *PDCD5 *in U2OS suggest that apoptosis is a target pathway for OS epigenetic drug intervention. These genes would be ideal markers for successful induction of demethylation in OS-derived cells, OS primary cells, and OS clinical trials.

## Methods

### Cell line culture and treatment

The human OS cell line U2OS was obtained from the American Type Culture Collection (ATCC) (Rockville, MD) and maintained in alpha-Minimum Essential Medium (alpha-MEM) supplemented with 10% heat inactivated Fetal Bovine Serum and 2 mM L-Glutamine. Treatment with decitabine was performed as described by Liang et al [39]. Briefly, 5 × 10^5 ^cells were plated in 56 cm^2 ^culture plates with 10 ml growth medium. 12 hours after plating they were treated with freshly prepared decitabine (Sigma Chemical Co., St Louis, MO) to a final concentration of 1 μM without changing the medium. The cells were harvested by trypsinization after 3 days of treatment, where the cells were portioned and used for total RNA extraction, DNA extraction, or Propidium Iodide (PI) staining for cell death by flow cytometery. A control (medium only) culture was maintained and processed over the same period of time under the same condition as the treated cells. To establish growth curves for U2OS cells with or without 1 μM decitabine, cells were plated at 5 × 10^5 ^cells/56 cm^2 ^culture plates with 4 mm^2 ^grids. The cells were allowed to attach to the surface of the plates for 12 hours before the start of the treatment. Adherent cells were counted in 2 independent cultures in multiple 4 mm^2 ^grids every 12 hours after plating and the experiment was repeated after culturing the cells for 5 passages. When cell growth was near confluent, the cells were trypsinized, re-suspended in growth medium (10% serum) and cell viability was determined using Vi-CELL™ XR (Beckman Coulter, Fullerton, CA) after Trypan Blue staining.

### Immunohistochemistry and image analysis

Xenograft tissue sections were de-paraffinized using xylene and re-hydrated in a series of alcohols. The tissue sections were then incubated at room temperature (RT) in 3% H_2_0_2 _in PBS for 10 minutes to inactivate endogenous peroxidase. Following incubation the slides were washed 3 times in PBS for 3 minutes each. Antigen retrieval was obtained by heating in a microwave at maximum heat for 20 minutes in Tris-EDTA buffer (10 mM Tris Base, 1 mM EDTA solution, 0.05% Tween 20, pH 9.0) and cooling for 20 minutes at RT. Slides were again washed 3 times in PBS for 3 minutes each. The slides were blocked (30 minutes in a humid chamber at RT) with serum to reduce non-specific binding. Serum was removed from the slides and the slides were then incubated with the primary antibody 5-methylcytidine (5-mc-Ab) (Eurogentec, San Diego, CA) at 1:500 dilution at 4°C overnight.

Following incubation the slides were washed 3 times in PBS for 3 minutes each. The slides were then incubated with a secondary antibody [Polyclonal rabbit anti-mouse immunoglobulins/biotinylated rabbit F(ab')2; Dako] for 30 minutes at RT, followed by 3 washes in PBS for 3 minutes each. The slides were then incubated with StreptABCComplex/HRP (Dako, Glostrup, Denmark) for 30 minutes in a humid chamber at RT, followed by 3 washes in PBS for 3 minutes each. A 3,3'diaminobenzidine (DAB) substrate (Vector Laboratories, Burlingame, CA) was used for detection and hematoxylin was used for counterstain. The slides were then dehydrated and mounted.

Whole sections from xenografts were scanned by ScanScope CS (Aperio technologies, Vista, CA). The slides were digitized to 20× magnification (~0.5 microns/pixel). Images were then viewed with Aperio's image viewer software (ImageScope), which allows performing quantitative analysis of stain intensity on snapshots from the sections. Five to ten ~0.3 mm^2 ^snapshots (each containing 3,000 to 5,000 cells) were analyzed per section using the following parameters: compression quality = 30, and color saturation threshold = 0.04. Positivity thresholds were150 to 220 = high positive, 100 to 150 = low positive, and 0 to 100 = negative. Descriptive analysis such as mean and standard deviation for 5-mc immunostaining intensity were calculated based on the percentage of positivity (total positivity/total negativity per snapshot). Comparison between control and decitabine-treated sections was done using the student t-test and p < 0.05 was considered significant.

### In vivo studies: U2OS xenograft and treatment

Six- to eight-week old male immune-deficient NOD-SCID and Rag-2M mice were bred and maintained by the Animal Resource Centre at the British Colombia Cancer agency, Vancouver, Canada. U2OS cells, in general, were considered as non-tumorigenic in mice while grafting the cells subcutaneously or orthotopically [17]. As such, in order to establish their xenografts, U2OS cells were grafted under the renal capsule, a site proven to be an excellent site for tumor engraftment [18-20]. Briefly U2OS cells were cultured in alpha-MEM and washed in growth medium containing 20% FBS. The viable cells were counted after trypan blue staining. 2 × 10^6 ^cells were pelleted, re-suspended and grafted beneath the renal capsule of adult male SCID mice as previously described [18]. After 5 months, a well-grown xenograft was selected for re-grafting to establish multiple stable U2OS xenografts under the kidney capsules of NOD-SCID mice (two per kidney per mouse). The re-grafted U2OS xenograft had a 100% take rate, with a doubling time of ~10 days and exponential growth phase starting after ~2 of tumor growth doubling. After 5 generations of tumor growth doubling the xenografts were surgically removed from the mice and cut into approximately 4 mm^3 ^portions then were re-grafted under the renal capsules of 6 male Rag-2M mice (4 grafts per mouse, 2 per kidney). Four weeks after grafting (~2–3 doubling of tumor growth), the host mice were divided into two groups. One group (3 mice) was given decitabine (2.5 mg/kg body weight) dissolved in saline (0.9% w/v NaCl), intraperitoneally [8] on days 29, 31 and 33. The other group (3 mice) was given saline alone as a treatment control over the same schedule. On day 37, mice from both groups were sacrificed. Tumor volumes were measured using a digital caliper, recorded and expressed in mm^3^, using the formula: volume (mm^3^) = (0.52) × length (mm) × width (mm) × height (mm). Data were presented as means ± Standard Deviation (SD) and student t-test was used to analyze the difference between the two treatment groups. The xenograft tissues were then snap frozen, or prepared in paraffin and sectioned according to standard procedures [18].

### Histopathological analysis and TUNEL assay

The tissues from the control (no treatment) and decitabine treated groups were paraffin embedded using routine protocols [18] and stained with hematoxylin and eosin. Sections were assessed blindly. Extracellular matrix was defined as eosinophilic osteoid-like material surrounding individual cells and small clusters of 3–5 cells, and the percentage of tumor with osteoid was then calculated. Mitotic counts were performed in areas with the highest mitotic rate, and ten high-powered fields (× 400) were counted per section.

*In situ *hybridization for terminal deoxynucleotidyl transferase-mediated nick end labeling (TUNEL) was performed on paraffin sections as recommended by the manufacturer (Ventana Medical Systems, Tucson, AZ). Scoring of the sections was performed using Simple PCI analytical software (Nikon, Tokyo, Japan). Sections were examined and the most intense areas of staining were photographed using a DXM1200 digital camera (Nikon) at a power of x200. The digital image was then scanned using the Simple PCI program and the numbers of positive and negative nuclei were obtained. Control and treatment images were all photographed at a uniform brightness, and all images were subjected to uniform binary image modification and size calibration prior to counting by Simple PCI. The positivity index was obtained by dividing the number of positive nuclei by the total number of nuclei (positive + negative). The number of nuclei counted was always over 1000, and ranged from 1100 to 2000. Positivity indices were compared by Student's t-test and a p-value of <0.05 was considered significant.

### Affymetrix expression analysis

Total RNA was extracted using the RNeasy kit (Qiagen, Germany) from duplicate experiments of U2OS cells at day 3 after treatment with 1 μM decitabine or medium alone (control). In each experiment RNA yields were pooled from two independent cultures per treatment arm to minimize experimental noise. For each case, 10 μg of RNA was labeled and hybridized to the Affymetrix HG-U133A GeneChips using the manufacturer's protocol (Affymetrix, Santa Clara, CA) by the Centre of Applied Genomics at the Hospital for Sick Children (Toronto, Canada). Data were extracted using the Microarray Suite (MAS) version 5.0 (Affymetrix) and linearly scaled to achieve an average intensity of 150 across each chip. The candidate gene list obtained from the MAS 5.0-extracted data was selected by eliminating genes that were not present in at least one experiment. The arrays were subjected to a pair wise comparison using MAS 5.0, with signal intensities from the no-treatment cells as the baseline. The statistical significance for the change of expression for each probe set between the decitabine treated and control was calculated by the MAS 5.0 software. The criteria for gene selection for real-time expression validation analysis was based on the statistically significant up-regulation (p < 0.0025) and fold change of ≥2 for expression after decitabine treatment. The gene list was annotated based on the NetAffx data-base [58] and further verified using the Human Genome Browser data base [59]. All the raw data for expression arrays is available in [60] under the series record number (GSE7454).

### In silico analysis of CpG-island association, gene annotation, and pathway enrichment

The criteria for a CpG-island was based on those outlined by Takai and Jones [61], where the GC ≥ 55%, Obs/Exp ≥ 0.65, and length > 300 bp which was reported to exclude most *Alu*-repetitive elements. We identified the genes that harbored CpG-island within a 2000 bp window upstream or downstream from the transcription start site based Human Genome Browser data base [59]. To be certain that there were no CpG island closer to the TSS and gene promoter regions, we submitted the sequences of interest (including a 2000 bp window upstream and downstream from TSS) to the CpG search engine available in reference [61] and verified that there was no CpG islands that are closer to TSS for the genes we tested. Up-regulated genes with CpG-island associations were further analyzed through the Microarray Literature-based Annotation tool MILANO [62] to look for evidence of epigenetic modifications in the literature. MILANO is a web-based tool that allows annotation of lists of genes derived from microarray results by user defined terms [62]. Using MILANO we searched for literature associations between our list of genes and the terms 'epigenetics', 'methylation' 'chromatin modification' 'cancer', and 'disease'. To identify the putative functional pathways for each gene list, we used the functional annotation enrichment tool. This tool utilizes the Gene Ontology database and uses GO Terms to identify enriched biological themes in the gene lists [63,63]. The Fisher Exact test was applied to determine the significance in the proportions of genes falling into a certain pathway in each gene list. We used this tool to look for enriched pathways of up- or down- regulated genes with CpG-island associations from the gene lists from the cell lines.

### Expression validation using reverse transcription and quantitative real-time PCR

Total RNA from xenografts was extracted using the TRIzol reagent method. 1 ml of TRIzol (Invitrogen, Osaka, Japan) was used for every 50–100 mg of tumor tissue and homogenized in an RNase free environment. Chloroform was then added (200 μl for each 1 ml TRIzol) and the samples were centrifuged at high speed for 15 minutes at 4°C. The aqueous layer was then transferred into a new tube and RNA was precipitated with iso-propanol followed by one wash using 70% ethanol. The RNA precipitate was then dissolved in 10–15 μl of RNAse free water and analyzed for quantity and quality using a spectrophotometer. A two-step reverse transcription-PCR procedure was performed. Total RNA was reverse transcribed using the GeneAmp kit (Applied Biosystems; ABI, Foster City, CA). 20 ng of the resulting cDNA was then used in the real-time PCR step. Six genes were tested by real-time PCR including: *growth arrest and DNA-Damage inducible, alpha *(*GADD45A*), *heat chock70KDA protein 9b *(*HSPA9B*), *parkc apoptosis wt1-regulatort *(*PAWR*), *programmed cell death 5 gene *(*PDCD5*), *nuclear factor of kappa light polypeptide gene enhancer in B-cells inhibitor, alpha *(*NFKBIA*), *tumor necrosis factor, alpha-induced protein 3 *(*TNFAIP3*). We used the TaqMan primers (ABI) for all the genes that we tested (primer information is provided the in [Additional file 5]). All real-time PCR assays were performed in triplicate in a 96-well plate using the 7900 Sequence Detector System (ABI) according to the manufacturer's protocol. Data analysis was performed using the Sequence Detector System (SDS) software (ABI) and the results were expressed as fold-change in relative mRNA expression level, calculated using the ΔΔCt method with β-actin (*ACTB*) as the reference gene and the non-treated cells as baseline. The validation was carried out on RNA from three replicate experiments of U2OS cells, three decitabine-treated U2OS xenograft tumors (Xeno- 1, 2 and 3), three no-treatment (control) U2OS xenograft tumors (Xeno- 4, 5, and 6) and three replicate experiments of NHOst.

### Quantitative-bisulfite pyrosequencing

Quantitative Bisulfite Pyrosequencing for CpG islands (Pyro Q-CpG) is a sequencing-based analysis of DNA methylation that quantifies multiple CpG sites per amplicon using Pyro Q-CpG software. 2 μg of DNA from the control and decitabine treatment were bisulfite-treated using the Zymo DNA Methylation Kit (Zymo Research, Orange, CA). Bisulfite-treated DNA was amplified by PCR then sequenced according to the manufacturer's protocol (Biotage, Kungsgatan, Sweden). The target sequences inside the CpG-islands of the candidate genes and the primer sequences are shown in [Additional file 5]. The percentage of C content (methylated alleles) versus T content (unmethylated alleles) is calculated by the Pyro-Q-CpG software for each CpG position in each sample. Analysis was performed on DNA samples from 3 replicate experiments of U2OS cells *in vitro *and six U2OS xenograft tumors; three decitabine treated (Xeno-1, Xeno-2 and Xeno-3), and three saline (control) treated (Xeno-4, Xeno-5 and Xeno-6). Universally methylated DNA was used as a methylation positive control. DNA isolated from early embryos (Biotage, Kungsgatan, Sweden) was used for methylation negative control. DNA from low-passage normal human osteoblasts (PromoCell, Germany) was used for experiment control.

## Competing interests

The author(s) declare that they have no competing interests.

## Authors' contributions

K A performed the *in vitro *treatment assays, the AffyChip expression assays, the AffyChip data analysis, the real-time expression assays and analysis *in vitro *and *in vivo*, the DNA methylation data analysis, the meth5-C data analysis and conceived and wrote the manuscript draft; G R S performed the histopathological analysis and TUNEL analysis; J B contributed to the writing of the paper; S H performed the immunostaining using the Methyl-C-Ab; M P contributed to the AffyChip data analysis; J-C C contributed to the mice experiments; H X and Y W performed the mice experiment; M Z and J A S conceived and contributed to the writing of the manuscript. All authors read and approved the final manuscript.

## Supplementary Material

Additional file 1 The effectiveness of demethylation following 72 hours treatment with 1 μM decitabine at the *SNRPN *gene locus. The imprinted *SNRPN *gene is located on human chromosome band 15q11.3 and alterations in DNA methylation at this locus are associated with individuals with the Prader-Willi and Angelman syndromes [64,65]. It was utilized in this study to confirm that decitabine treatment reduced DNA methylation in U2OS cells. A) Schematic figure of the *SNRPN *gene showing the probe location relative to *Not*I and *Xba*I cutting sites. *Not*I is a methyl-sensitive restriction endonuclease that will only cut its recognition sequence when unmethylated. B) Samples 1 and 2 are controls and samples 3 and 4 were treated with decitabine for 3-days. Left panel shows the autoradiogram of the restriction digest of DNA samples 1–4 on a 0.8% agarose gel. Right panel is Southern blot showing an increase of 63% of the 0.9 kb *Not*I product as a result of decitabine treatment (63 % loss of CpG methylation at the locus in U2OS). Click here for file

Additional file 2 Effect of decitabine on U2OS xenografts size (raw data). Raw data measurements of 12 control tumors and decitabine treated tumors expressed as in tumor volumes estimated using this formula: **volume (mm^3^) = (0.52) × (length mm) × (width mm) × (height mm)**. L = Length, W = width, H = height. SD = standard deviation. Click here for file

Additional file 3 Expression fold change as detected in the Affy experiments for U2OS (U2OS1 and 2). All listed genes had change p-value < 0.0025 in the duplicate experiments. FC = fold change. I = increase. Click here for file

Additional file 4 Methylation % detected by Pyro-Q-CpG (raw data). Multiple CpG positions were tested for each gene for all samples and indicated is the methylation percentage in all experiments. NT and (CT) = no-treatment (control). TR = decitabine treated. NHOst = Normal human osteoblasts. Click here for file

Additional file 5 Additional methodology. Detailed description of the methods and materials used in the experimental approach. Click here for file
