# Supplementary figures and images for: Modulation by decitabine of gene expression and growth of osteosarcoma U2OS cells in vitro and in xenografts: Identification of apoptotic genes as targets for demethylation
Source: Cancer Cell Int. 2007 Sep 10;7:14. doi: 10.1186/1475-2867-7-14 (PMC2034371; doi:10.1186/1475-2867-7-14)

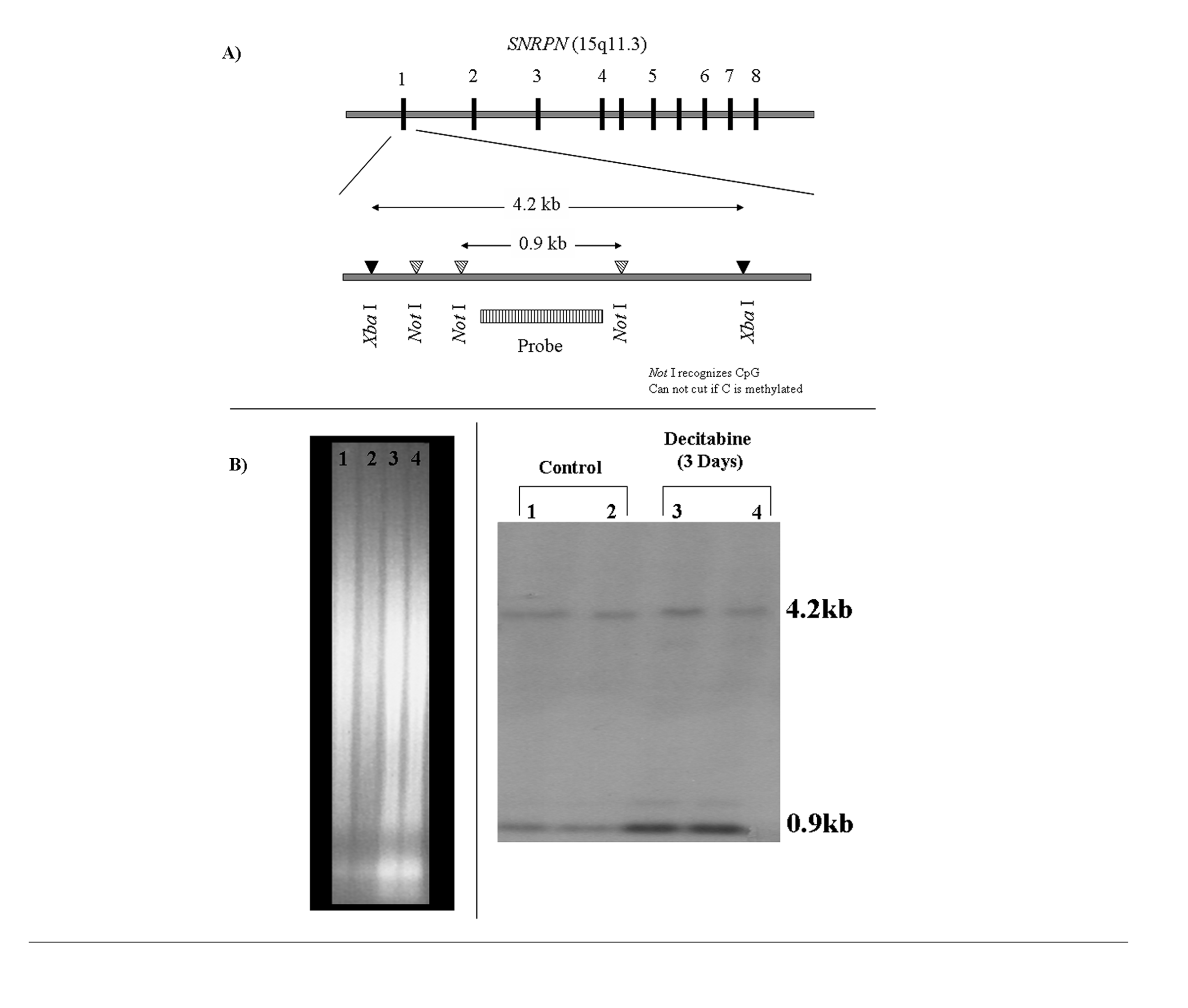

Supplement: Additional file 1 — The effectiveness of demethylation following 72 hours treatment with 1 μM decitabine at the SNRPN gene locus. The imprinted SNRPN gene is located on human chromosome band 15q11.3 and alterations in DNA methylation at this locus are associated with individuals with the Prader-Willi and Angelman syndromes [64,65]. It was utilized in this study to confirm that decitabine treatment reduced DNA methylation in U2OS cells. A) Schematic figure of the SNRPN gene showing the probe location relative to NotI and XbaI cutting sites. NotI is a methyl-sensitive restriction endonuclease that will only cut its recognition sequence when unmethylated. B) Samples 1 and 2 are controls and samples 3 and 4 were treated with decitabine for 3-days. Left panel shows the autoradiogram of the restriction digest of DNA samples 1–4 on a 0.8% agarose gel. Right panel is Southern blot showing an increase of 63% of the 0.9 kb NotI product as a result of decitabine treatment (63 % loss of CpG methylation at the locus in U2OS). [file 1475-2867-7-14-S1.tiff]
