# Supplementary material for: Modulation by decitabine of gene expression and growth of osteosarcoma U2OS cells in vitro and in xenografts: Identification of apoptotic genes as targets for demethylation
Source: Cancer Cell Int. 2007 Sep 10;7:14. doi: 10.1186/1475-2867-7-14 (PMC2034371; doi:10.1186/1475-2867-7-14)
Supplement: Additional file 5 — Additional methodology. Detailed description of the methods and materials used in the experimental approach. [file 1475-2867-7-14-S5.doc]

Supplementary Materials and Methods

*Cell line culture and treatment*

The human OS cell line U2OS was purchased from the American Type Culture Collection (Rockville, MD) and maintained in Minimum Essential Medium (MEM) Alpha (alpha-MEM) supplemented with 10% heat inactivated Fetal Bovine Serum and 2 mM L-Glutamine and cultured in an incubator at 37°C in the presence 5% CO2. Cell treatment was described elsewhere (Liang et al., 2002). Briefly, cells were plated at 5X10^5^ cells/75 mm^2^ culture flasks and were treated after 12 hours of plating with freshly prepared 5-Aza-2’-deoxycytidin (decitabine) (Sigma Chemical Co., St Louis, MO) dissolved in culture medium at a final concentration of 1µM. Cells were harvested by trypsinization after 3 days of treatment and portions of the cells were used for total RNA extraction, DNA extraction, or PI staining for death detection using flowcytometery. ‘Medium alone’ cells were cultured over the same period of time in the same conditions as the decitabine treated cells and were harvested, processed and analyzed in the same time following the same procedures. At day 3 after treatment the cells were portioned and used for total RNA extraction, DNA extraction, or Propidium Iodide (PI) staining for cell death by flow cytometery. For PI staining the cells were detached from culture surface by trypsinization homogenized with culture medium with 10% heat inactivated fetal bovine serum then washed with PBS twice before they were incubated with 10 µl PI (50g/ml)/ 1x10^5^ cells. After 15 minutes incubation in the dark the cells were run in a flowcytometer and the percentage of PI stained cells was determined and a comparison between control and no-treatment was performed. For each run we used un-treated cells without PI staining to calibrate the gate for U2OS cells then.

The growth curves for U2OS cells with or without 1uM decitabine were done by counting adherent cells of 2 independent cultures every 12 hours after seeding (seeding density 5X10^5^) on 100mm plates with 4mm^2^ grids. Multiple random grids were counted at each time point for a duplicate experiment. The final cell count was done after harvesting the cells by trypsinization and dying with Trypan Blue using an automated cell counter, Vi-CELL^TM^ XR (Beckman Coulter, Fullerton, CA). The experiment was repeated after growing U2OS for 5 passages to ensure that sub-culturing did not affect the cell growth kinetics.

*Immunohistostaining and image analysis*

Xenograft tissue sections were de-paraffinized using xylene and re-hydrated in a series of alcohols. The tissue sections were then incubated at room temperature (RT) in 3% H_2_0_2_ in phosphate buffered saline (PBS) for 10 minutes to inactivate endogenous peroxidase. Following incubation the slides were washed 3 times in PBS for 3 minutes each. Antigen retrieval was obtained by heating in a microwave at maximum heat for 20 minutes in Tris-EDTA Buffer (10mM Tris Base, 1mM EDTA Solution, 0.05% Tween 20, pH 9.0) and cooling for 20 minutes at RT. Slides were again washed 3 times in PBS for 3 minutes each. The slides were blocked (30 minutes in a humid chamber at RT) with serum to reduce non-specific binding. The slides were then incubated with primary antibody 5-methylcytidine (5-mc-Ab) at 1:500 dilution (Eurogentec) at 4°C overnight.

Following incubation the slides were washed 3 times in PBS for 3 minutes each. The slides were then incubated with secondary antibody [Polyclonal rabbit anti-mouse immunoglobulins/biotinylated rabbit F(ab’)2; Dako] for 30 minutes at RT, followed by 3 washes in PBS for 3 minutes each. The slides were then incubated with StreptABC-Complex/HRP (Dako) for 30 minutes in a humid chamber at RT, followed by 3 washes in PBS for 3 minutes each. A 3,3’diaminobenzidine (DAB) substrate (Vector Laboratories) was used for detection and hematoxylin was used for counterstain. The slides were then dehydrated and mounted.

Whole sections from xenografts were scanned by ScanScope CS ([Aperio technologies](http://www.aperio.com/), CA, USA). The slides were digitized to 20x magnification (~ 0.5 microns/pixel). Images were then viewed with Aperio's image viewer software (ImageScope), which allows performing quantitative analysis of stain intensity on snapshots from the sections. Five to ten ~ 0.3 mm^2^ snapshots (each containing 3,000 to 5,000 cells) were analyzed per section using the following parameters: compression quality = 30, and color saturation threshold = 0.04. Positivity thresholds were150 to 220 = high positive, 100 to 150 = low positive, and 0 to 100 = negative. Descriptive analysis such as mean and standard deviation for 5-mc immunostaining intensity were calculated based on the percentage of positivity (total positivity/total negativity per snapshot). Comparison between control and decitabine-treated sections was done using the student t-test test and p< 0.05 was considered significant.

*U2OS xenograft and treatment*

Six- to eight-week old male NOD-SCID and Rag-2M mice were bred by the BC Cancer Research Centre Animal Resource Centre, BC Cancer Agency, Vancouver, Canada. Decitabine was purchased from Sigma and prepared into 50 mg/ml stock solution in normal saline (0.9% w/v NaCl) and stored in -20°C. U2OS cells were maintained as described above. When cultures reached 70- 80% growth, the cells were released from tissue culture plates with trypsinization, washed in growth medium containing 20% FBS, and viable cells were counted after Trypan Blue stain on a hematocytometer. 2x10^6^ cells were pelleted and re-suspended in 50 lof-neutralized type-1 rat tail collagen prepared as described previously (Wang et al., 2005). Collagen gels containing 2x10^6^ cells per gel were allowed to set at 37°C for 15 min and were then covered with growth medium and cultured overnight. 12 gels containing U2OS cells were grafted beneath the renal capsule of adult immune-deficient male SCID mice. After 5 months of grafting, one graft out of 12 showed significant tumor growth. The rest of grafts were viable, but small.

The first well-grown xenograft was selected for re-grafting to establish multiple stable U2OS xenografts under the kidney capsules (two per kidney per mouse). After 5 generations of tumor growth doubling in mice, the established U2OS xenografts under the renal capsule of SCID mice had 100% take rate with doubling time of 8 days. U2OS tumor xenografts were obtained from SCID mice and cut into pieces of ~4 mm^3^ and 48 tumor tissue pieces were re-grafted under the renal capsule of 6 male Rag-2M mice (4 grafts per mouse, 2 each kidney). 4 weeks after grafting (about 2-3 doubling of tumor growth), the host mice were divided into two groups. One group of 3 mice was given decitabine (2.5 mg/kg body weight) interperitoneally on day 29, 31 and 33. The other group of 6 mice was given saline (control) at the same schedule. At day 37, mice from both groups were sacrificed. Tumor volumes were recorded using a digital caliber and expressed in mm^3^, using the formula: volume (mm^3^) = (0.52) X (length) X (width) X (height) (in mm). Data were presented as means ± S.D. Snap frozen tissue, O.C.T block and paraffin blocks from each graft were prepared. A duplicate experiment was done independently and showed similar observations.

*Histopathological Analysis*

Tumor tissue was harvested and fixed in 10% neutral buffered formaldehyde for 24 hours. The tissues then underwent routine processing for paraffin embedding using routine protocols. Sections were cut at 4 µm mounted on glass slides and stained with Hematoxylin and Eosin (H&E). The slides were assessed for the presence of tumor tissue, and those sections with no tumor tissue were excluded from the analysis, and subsequent analysis was done blindly. Extracellular matrix was defined as eosinophilic osteoid-like material surrounding individual cells and small clusters of cells, and the percentage of tumor with osteoid was then calculated. Mitotic counts were performed in areas with the highest mitotic rate in 9 sections from each treatment group, and ten high-powered fields (x 400) were evaluated per section.

*In situ* hybridization for terminal deoxynucleotidyl transferase-mediated nick end labeling (TUNEL) was performed on 4-µm thick sections placed onto saline-coated glass slides. Apoptotic cells were detected using the TUNEL assay for in situ end labeling, adapted to an automated in situ hybridization instrument (Discovery; Ventana Medical Systems). Before staining, the sections were blocked for endogenous peroxidase with subsequent protease I (Ventana Medical Systems) digestion for 12 minutes and a biotin block. The assay uses recombinant terminal deoxynucleotidyl transferase (Invitrogen, Carlsbad, CA) to add homopolymer tails to the 3' ends of cleaved DNA, characteristic in cells undergoing programmed cell death. Biotin 16-dUTP (Roche Diagnostics, Mannheim, Germany) was used to label this reaction. Colorimetric visualization using avidin-horseradish peroxidase and the 3, 3’-diaminobenzidine detection method was performed. The counter stain of choice was Hematoxylin.

Scoring of the sections was performed using Simple PCI analytical software (Nikon). Sections were examined and the most intense areas of staining were photographed using a DXM1200 digital camera (Nikon) at a power of x200. The digital image was then opened using the Simple PCI program and the numbers of positive and negative nuclei were obtained. The control and treatment images were all photographed at a uniform brightness, and all images were subject to uniform binary image modification and size calibration prior to counting by Simple PCI. The positivity index was obtained by dividing the number of positive nuclei by the total number of nuclei (positive + negative). The number of nuclei counted was always over 1000, and ranged from 1100 to 2000. Positivity indices were compared by Student’s t-test and a p-value of <0.05 was considered significant.

*Expression analysis using Oligonucleotide array*

RNA yields were pooled from two independent experiments that were performed simultaneously (with or without 1uM decitabine) a duplicate experiment was performed in the same manner. Total RNA was extracted using the RNeasy kit (Qiagen, Germany), labeled and hybridized to Affymetrix Genechip HG-U133A arrays (Affymetrix, Santa Clara, CA) by the Center of Applied Genomics at the Hospital for Sick Children (Toronto, Canada), according to the manufacturer’s protocol. Data were extracted using Microarray Suite (MAS) version 5.0 (Affymetrix) and linearly scaled to achieve an average intensity of 150 across each chip. The candidate gene list obtained from MAS 5.0-extracted data was selected by eliminating genes that were not present in both experiments. The arrays were subjected to a pair wise comparison using MAS 5.0 with un-treated (control) signal intensities as the baseline for each cell line. The statistical significance for up-regulation of expression for each probe set between treated and un-treated cells was calculated by MAS5.0 software. Genes with Statistically significant up-regulation of expression (p<0.0025) were then extracted (88 genes) and found to have a fold-change that ranged from 1.2 to 5.6. Criteria for gene selection for subsequent analysis were based on statistical significance (p<0.0025) for expression up-regulation after decitabine treatment and fold change of ≥ 2. The gene list was annotated based on the NetAffx data-base ([**http://www.affymetrix.com/analysis /index.affx**](http://www.affymetrix.com/analysis /index.affx)) and further verified using the Human Genome Browser data base ([**http://genome.ucsc.edu/**](http://genome.ucsc.edu/))

*In silico analysis of CpG-island association, gene annotation, and pathway enrichment*

The criteria for a CpG-island were based on Takai and Jones: GC ≥ 55%, Obs/Exp ≥ 0.65, and length > 300 bp, at ([**http://cpgislands.usc.edu**](http://cpgislands.usc.edu/)) which was reported to exclude most *Alu*-repetitive elements (Takai & Jones, 2003). We further extracted the genes that harbor CpG-island within a 2000 bp window upstream or downstream from transcription start site using the Human Genome Browser (http://genome.ucsc.edu/). Up-regulated genes with CpG-island association were further run through the Microarray Literature-based Annotation tool (MILANO) (http://milano.md.huji.ac.il/) to look for evidence of epigenetic modifications in the literature. MILANO is a web-based tool that allows annotation of lists of genes derived from microarray results by user defined terms (Rubinstein & Simon, 2005). Using MILANO we searched for literature association between our list of genes and ‘epigenetics’, ‘methylation’ ‘chromatin modification’ ‘cancer’, and ‘disease’. To identify the candidate functional pathways for each gene list we used the functional annotation enrichment tool at ([**http://niaid.abcc.ncifcrf.gov/**](http://niaid.abcc.ncifcrf.gov/)). This tool utilizes the Gene Ontology database and uses GO Terms to identify enriched biological themes in gene lists (Dennis et al., 2003) (Dennis et al., 2003). It applies the Fisher Exact test to determine the significance of the proportions of genes falling into a certain pathway in each gene list. We used this tool to look for enriched pathways of up- or down- regulated genes with CpG-island association of gene lists from the cell lines.

*Expression validation using Reverse Transcription and Quantitative Real-Time PCR*

Two-step reverse transcription-PCR was conducted in this study. Total RNA form same samples, which were used for the AffyChip experiment were used to synthesize cDNA using the GeneAmp kit (Applied Biosystems ABI, Foster City, CA). Reverse transcription was done in a 20 µl volume containing 2 µg total RNA, 2.5 mM MgCl2, 1 mM dNTPs, 10 U RNase inhibitor, 15 U Reverse transcriptase and 25 µM random hexamers. The reactions were incubated at 42 C for 15 minutes, 99 C for 5 minutes and 5 C for 5 minutes. 20 ng of the resulting cDNA was then used in the real-time PCR step. We first used the TaqMan Endogenous Control Plate (Applied Biosystems ABI, Foster City, CA) to test the expression of 12 human housekeeping genes across our samples. The human β-Actin (*ACTB*) showed the lowest level of expression and least variability across the samples, hence we chose *ACTB* to work as the reference in the real-time experiment. In all the genes we tested, we used the TaqMan (ABI, USA) a list of the primers is shown below. Each reaction was 25 µl in volume and it contained 2 µl (20 ng) cDNA, 12.5 µl of 2x PCR Master Mix (ABI, CA), 1.25 µl of 20x Primer and 9.25 µl RNase Free water. All real-time PCR assays were done in triplicates on a 384-well plate using the 7900 Sequence Detector System (ABI) under these conditions: 50 C for 2 minutes, 95 C for 10 minutes, 40 cycles of 95 C for 15 seconds and 60 C for 60 seconds. Data analysis was done using the Sequence Detector System (SDS) software (ABI, CA) according to manufacturer’s manual. Normal osteoblasts (from low passage cultures) and Universal RNA (stratagene, La Jolla, CA) were used as controkes for all experiments and relative mRNA expression level was calculated using the ∆∆Ct method using ACTB as reference gene and un-treated cells as baseline.

| Gene | TaqMan assay# |
| --- | --- |
| ***GADD45A*** | Hs00169255_m1 |
| ***HSPA9B*** | Hs00269818_m1 |
| ***TNFAIP3*** | Hs00234713_m1 |
| ***PAWR*** | Hs00169332_m1 |
| ***NFKBIA*** | Hs00153283_m1 |
| ***PDCD5*** | Hs00270435_m1 |
| beta-Actin | 4352935E |

TaqMan (Applied Biosystems ABI, Foster City, CA) assays used for Real-Time PCR

*Quantitative-bisulfate Pyrosequencing*

Quantitative Bisulfate Pyrosequencing for CpG islands (Pyro Q-CpG) is a sequencing-based analysis of DNA methylation that quantify multiple, and consecutive CpG sites individually. 2ug of DNA from control and decitabine treatment was bisulfate treated using the Zymo DNA Methylation Kit (Zymo research, Orange, CA). Bisulfate treated DNA is eluted in10 µl volume and 2 µl (containing 200 ng DNA) of it was used for each PCR. The PCR amplification and sequencing reaction protocol is according to the manufacture’s guide (Biotage, Kungsgatan, Sweden). The PCR is performed with one of the PCR primers biotinylated to convert the PCR product to single-stranded DNA templates. The target sequences inside the CpG-islands of the candidate genes and the primer sequences are shown below. Sequencing reaction is then achieved using a sequencing primer and an automated sequence reading indicates the quantity of C and T in a form of peaks. The C content are proportional to the number of methylated alleles at each CpG dinucleotide. C content (methylated allells) is the expressed as percentage of the alleles with C in each CpG site. Non-treated cells were compared to decitabine treated cells using universally methylated DNA as positive control for methylation and DNA isolated from early embryos (Biotage, Kungsgatan, Sweden) for negative control. The data is presented as percentage of methylation before and after treatment at each tested CpG site (Mirmohammadsadegh et al., 2006).

**PCR and Pyrosequencing Assay Design**

Red = the PCR primers.

**Underlined bold** = the Pyrosequencing primers.

For Genome map view see Figure 4

**1- *GADD45A***

Original sequence:

**CTGGCAGCCTGTGGCAGGGGCACT**CTCGGGACTTCTCACGGGACGCCCGGTC**CTTGGGCGTGCAGGGGTCAT**GGGGGGTGACGGGGCCGCGGGAGCGCCGGGTTTTCGTAGAGCCCAGGTGCGCGGTGGTGCTTGCATTCGAGAGGGAGGGGCGTGGTACCGGACGAGGGGGGCGGCGATGGCCCCGAGGGCACCGGGGCTGACGGGACCCCTCGCCCTTGCCCGCGTGT**AGGATGGATAAGGTGGGGGAT**

Bisulfite converted PCR amplicon sequence:

**TTGGTAGTTTGTGGTAGGGGTATT**TTYGGGATTTTTTAYGGGAYGTTYGGTT**TTTGGGYGTGTAGGGGTTAT**GGGGGGTGAYGGGGTYGYGGGAGYGTYGGGTTTTYGTAGAGTTTAGGTGYGYGGTGGTGTTTGTATTYGAGAGGGAGGGGYGTGGTATYGGAYGAGGGGGGYGGYGATGGTTTYGAGGGTATYGGGGTTGAYGGGATTTTTYGTTTTTGTTYGYGTGT**AGGATGGATAAGGTGGGGGAT**

**2- *HSPA9B***

Original sequence:

**ACCTCCAACCACGTGGGGTGAGGGGCGGGGGTTGGTCACT**GCGGCCCAGAGGCCCGCTCTTCCGCTGAGGCGCCGCCCGCGCCGCCTGACCAGAGGATACTCGACTGCCGCTCCCGGAGCCAGGGCGTACAGGTAATATGCTCCGCAGC**ATGATGGTTGGAGAAAGCCTGCC**

Bisulfite converted PCR amplicon sequence:

**ATTTTTAATTAYGTGGGGTGAGGGGYGGGGGTTGGTTATT**GYGGTTTAGAGGTTYGTTTTTTYGTTGAGGYGTYGTTYGYGTYGTTTGATTAGAGGATATTYGATTGTYGTTTTYGGAGTTAGGGYGTATAGGTAATATGTTTYGTAGT**ATGATGGTTGGAGAAAGTTTGTT**

**4-** ***PDCD5***

Original Sequence:

GGGCCTGGATCCAAGCACAAT**CTCAGCTTTTGGAGCCAGCA**GCCGGACCGAATTCAGTTTTTCCGGAGTTCAGTTGTTATGACCCAAGTTTAGCCGACAGGTATTGAGGGTCCGCTGTGTTCCCGGTGCTGAGAGCGCGGCAGGGTCGGAGGAACGCTTGCCCCGCCGGGATGACAGGGAGGGGCCGCCT**GGGAAGCTTGGATGGATCACA**

Bisulfite converted PCR amplicon sequence:

GGGTTTGGATTTAAGTATAAT**TTTAGTTTTTGGAGTTAGTA**GTYGGATYGAATTTAGTTTTTTYGGAGTTTAGTTGTTATGATTTAAGTTTAGTYGATAGGTATTGAGGGTTYGTTGTGTTTTYGGTGTTGAGAGYGYGGTAGGGTYGGAGGAAYGTTTGTTTYGTYGGGATGATAGGGAGGGGTYGTTT**GGGAAGTTTGGATGGATTATA**

**5- *PAWR***

Original Sequence:

GGGGGCGG**GGCCTCACTCTGCGATATAACTC**GGGTCGCGCGGCTCGCGCAGGCCGCCACCGTCGTCCGCAAAGCCTGAGTCCTGTCCTTTCTCTCTCCCC**GGACAGCATGAGCTTCACCACTCGCTCCA**

Bisulfite converted PCR amplicon sequence:

GGGGGYGG**GGTTTTATTTTGYGATATAATT**YGGGTYGYGYGGTTYGYGTAGGTYGTTATYGTYGTTYGTAAAGTTTGAGTTTTGTTTTTTTTTTTTT**TTYGGATAGTATGAGTTTTATTATTYGTTTTA**

**References:**

Dennis G, Jr., Sherman BT, Hosack DA, Yang J, Gao W, Lane HC and Lempicki RA. (2003). *Genome Biol*, **4,** P3.

Liang G, Gonzales FA, Jones PA, Orntoft TF and Thykjaer T. (2002). *Cancer Res*, **62,** 961-6.

Mirmohammadsadegh A, Marini A, Nambiar S, Hassan M, Tannapfel A, Ruzicka T and Hengge UR. (2006). *Cancer Res*, **66,** 6546-52.

Rubinstein R and Simon I. (2005). *BMC Bioinformatics*, **6,** 12.

Takai D and Jones PA. (2003). *In Silico Biol*, **3,** 235-40.

Wang Y, Revelo MP, Sudilovsky D, Cao M, Chen WG, Goetz L, Xue H, Sadar M, Shappell SB, Cunha GR and Hayward SW. (2005). *Prostate*, **64,** 149-59.
